# A Cold-Footed Lady

**Published:** 1886-03

**Authors:** 


### A COLD-FOOTED LADY.

Madam, allow me to prescribe for you. I have had a long experience in the management of delicate women, and believe I can give you some important advice. For the present I prescribe only for your feet:

First—Procure a quantity of woolen stockings, not such as you buy at the store under the name of lamb's wool that you can read a newspaper through, but the kind that your Aunt Jerusha in the country knits for you, that will keep your feet dry and warm in spite of wind and weather.

Second—If you want to be thorough, change them every morning, hanging the fresh ones by the fire during the night.

Third—Procure thick calf skin boots, double uppers and triple soles, and wear them from the first of October to the first of May. Make frequent applications of some good oil blacking.

Fourth—Avoid rubbers altogether, except a pair of large rubber boots, which may be worn for a little time through snow drifts or a flood of water.

Fifth—Hold the bottoms of your feet in cold water a quarter of an inch deep just before going to bed two or three minutes, and then rub them hard with rough towels and your naked hands.

Sixth—Now, madam, go out freely in all weathers, and, believe me, not only will your feet enjoy a good circulation, but as a consequence of the good circulation in the lower extremities, your head will be relieved of all its fullness, and your heart of its palpitations. Your complexion will be greatly improved, and your health made better in every respect.

